# Effects of priming exercise on oxygen uptake kinetics, muscle oxygenation, and metabolic load in trained anaerobic athletes: a randomized crossover trial

**DOI:** 10.7717/peerj.21221

**Published:** 2026-04-28

**Authors:** Jing Qing, Yingying Hou, Hanyue Zhang, Tingting Sun, Xiao Jia, Zhenxing Kong

**Affiliations:** 1 Key Laboratory of Exercise and Physical Fitness, Ministry of Education, Beijing Sport University, Beijing, China; 2 School of Physical Education, Northeast Normal University, Jilin, China

**Keywords:** Priming exercise, Oxygen uptake kinetics, Muscle oxygenation, Blood flow restriction, Anaerobic athletes

## Abstract

**Background:**

This study aimed to compare the acute and residual physiological responses elicited by three different priming exercise strategies, including high-intensity priming exercise (HIPE), low-intensity priming exercise (LIPE), and low-intensity priming exercise with blood flow restriction (LIPE-BFR), on oxygen uptake kinetics, muscle oxygenation, and metabolic load during severe-intensity cycling in trained anaerobic athletes.

**Methods:**

Sixteen trained anaerobic athletes completed a randomized crossover protocol with three priming conditions (HIPE, LIPE, and LIPE-BFR) in a randomized order. The experimental protocol began with an incremental exercise test to determine maximal oxygen uptake (VO_2_max), followed by five submaximal constant-load cycling trials to derive individual power–VO_2_ regression equations. The three priming interventions included HIPE, LIPE, and LIPE-BFR. Key physiological parameters, including pulmonary oxygen uptake kinetics, muscle oxygen saturation (SmO_2_), accumulated blood lactate (ABLa) concentration, accumulated oxygen uptake (AVO_2_), and accumulated oxygen deficit (AOD), were assessed to quantify oxygen utilization and metabolic demand across sessions.

**Results:**

Compared with HIPE, both LIPE and LIPE-BFR induced significantly lower amplitude (A) and longer time delay (TD), along with reduced reduced minimum SmO_2_ (SmO_2_min) and steady-state SmO_2_ (SmO_2_ss) during exercise (*P* < 0.05). HIPE elicited greater metabolic responses, including higher AVO_2_, AOD, and ABLa. Significant correlations were observed between VO_2_kinetics and metabolic demand, as well as between SmO_2_-derived indices and ABLa (*P* < 0.05). However, no significant differences were found among the three conditions in VO_2_ kinetics or metabolic outcomes during the subsequent severe-intensity cycling bout (*P* > 0.05), except for a higher VO_2_ baseline in the HIPE group (*P* < 0.05).

**Conclusion:**

Although the three priming strategies elicited distinct acute physiological responses, their residual effects on oxygen uptake kinetics and metabolic outcomes during subsequent severe-intensity exercise were largely comparable. Future studies should further explore alternative priming protocols and refine experimental designs to better clarify the effects of priming.

## Introduction

The ability to rapidly uptake and efficiently utilize oxygen during high-intensity exercise is a key determinant of metabolic efficiency, exercise tolerance, and athletic performance (Barstow, 1994; Gerbino, Ward & Whipp, 1996). Oxygen uptake kinetics (VO_2_ kinetics) describe the dynamic adjustments in oxygen transport and utilization from pulmonary ventilation and circulatory delivery to muscular extraction at exercise onset (Burnley & Jones, 2007). Key parameters include time delay (TD), amplitude (A), and time constant (Tau) (Xu & Rhodes, 1999). Delayed VO_2_ responses lead to increased accumulated oxygen deficit (AOD), earlier recruitment of anaerobic energy systems, accelerated blood lactate accumulation, and increased metabolic strain, ultimately contributing to premature fatigue and impaired performance (Goulding et al., 2021). As a result, strategies to accelerate VO_2_ kinetics and improve O_2_ delivery–utilization matching have become a central focus in exercise physiology and performance optimization.

Priming exercise—short bouts of preparatory physical activity—has been widely applied to enhance subsequent oxygen uptake responses and metabolic readiness (Goulding, Burnley & Wüst, 2023). Among the established modalities, high-intensity priming exercise (HIPE) involves brief exercise above the lactate threshold and is thought to activate oxidative enzymes, increase muscle perfusion, and reduce the time constant and slow component of VO_2_, thereby improving aerobic energy contribution (Sousa et al., 2014). However, the high metabolic load of HIPE may also induce neuromuscular fatigue or deplete phosphagen stores, limiting its applicability in some contexts (Endo et al., 2007). More recently, low-intensity priming exercise with blood flow restriction (LIPE-BFR) has gained attention. By applying external compression to induce localized hypoxia under low mechanical load, LIPE-BFR aims to replicate some physiological effects of HIPE—such as muscle deoxygenation and capillary recruitment—without excessive systemic strain (Salzmann, Sanchez & Borrani, 2021).

Although initial studies have applied priming strategies in anaerobic or strength-oriented athletes, the evidence remains fragmented, with inconsistencies in training status, exercise mode, and measurement approaches (Harrison et al., 2019). Consequently, it remains unclear whether different priming modalities can effectively enhance oxygen uptake kinetics and metabolic responses in trained anaerobic athletes. In anaerobic athletes, particularly sprinters, the physiological demands differ greatly from those of endurance athletes. Sprinters perform repeated high-intensity efforts with minimal recovery, which places a unique strain on anaerobic energy systems. These athletes rely heavily on short-duration, high-intensity energy output and may benefit from priming strategies that enhance oxygen uptake kinetics and metabolic efficiency to mitigate metabolic fatigue and optimize recovery between sprints (Pereira et al., 2025).

In this context, the present study compared three priming strategies—low-intensity priming (LIPE), high-intensity priming (HIPE), and low-intensity priming with blood flow restriction (LIPE-BFR)—and examined their effects on subsequent severe-intensity cycling in National First-Class short-distance sprinters. Oxygen uptake kinetics, muscle oxygen saturation (SmO_2_), and metabolic indicators—including AVO_2_, AOD, and accumulated blood lactate (ABLa)—were simultaneously assessed to provide a comprehensive evaluation of each priming modality. Based on previous literature, we hypothesized that (1) HIPE would accelerate VO_2_ kinetics relative to LIPE, (2) LIPE-BFR would produce comparable improvements to HIPE despite lower mechanical load, and (3) both HIPE and LIPE-BFR would reduce oxygen deficit and metabolic strain compared with LIPE.

## Materials & Methods

### Participants

A total of 16 athletes were recruited for this study, all of whom were certified National First-Class short-distance sprinters (100–200 m) in China (Mckay et al., 2022). The sample consisted of eight males and eight females, and detailed demographic characteristics are presented in Table 1. Given the limited sample size, no sex-based subgroup analyses were performed in this study. All participants were non-smokers and had a normal body weight, with no obesity or overweight conditions. Female participants were not using hormonal contraceptives and reported regular menstrual cycles (28 ± 2 days). The experimental protocol was reviewed and approved by the Sports Science Experiment Ethics Committee of Beijing Sport University (approval number: 2025151H). The study was conducted between March 1 and May 31, 2025, encompassing participant recruitment, preliminary screening, and all experimental testing sessions. Prior to data collection, all procedures, potential risks, and testing requirements were thoroughly explained by the lead investigator, and written informed consent was obtained from each participant. The research complied with the ethical principles of the Declaration of Helsinki.

**Table 1:** Baseline characteristics of all participants.

Variables	Participants (*n* = 16)
Age (years)	20.31 ± 1.04
Height (cm)	173.81 ± 7.81
Body weight (kg)	63.45 ± 8.93
BMI (kg/m^2^)	21.18 ± 1.71
%body fat	13.21 ± 4.29
VO_2max_ (mL/kg/min)	45.24 ± 4.87
Power at VO_2max_ (W)	238 ± 43
VT_1_(mL/kg/min)	22.70 ± 3.09
Power at VT_1_ (W)	99 ± 27
VT_2_ (mL/kg/min)	36.92 ± 4.27
Power at VT_2_ (W)	172 ± 32
Training experience (years)	4.41 ± 1.46

### Experimental design

Sixteen trained anaerobic athletes completed a standardized testing protocol over an eight-week period. The protocol included an incremental exercise test, constant-load cycling tests, and three separate priming interventions. To minimize fatigue effects, all testing sessions were spaced at least 48 h apart and scheduled at consistent times of day to reduce circadian variation. Although their primary sport is running, a cycle ergometer was used for all priming and testing procedures to ensure precise workload control, safe and repeatable high-intensity transitions, and consistent measurement of oxygen uptake kinetics, which is more difficult to standardize during treadmill running.

During the first laboratory visit, body composition was assessed using a multi-frequency bioelectrical impedance analyzer (InBody 230; InBody, Seoul, Korea). Participants then completed an incremental cycling test to determine maximal oxygen uptake (VO_2_max) and individual power thresholds, which were used to prescribe subsequent constant-load and priming intensities. The constant-load phase included five submaximal trials performed at intensities near the ventilatory threshold to establish a linear relationship between power output and oxygen uptake. In the main experimental phase, each participant completed three severe-intensity cycling trials, each preceded by a different priming strategy in randomized order:LIPE, HIPE, and LIPE-BFR. The cycling task following each priming intervention was identical across all conditions to ensure that the priming modality was the only experimental variable. The subsequent test consisted of a 10-min severe-intensity cycling bout performed at a fixed workload determined as 0.4 × (Pmax − VT_1_) + VT_1_, corresponding to approximately 110% of critical power.

All tests were conducted in a temperature-controlled laboratory (18 °C–21 °C) using a calibrated cycle ergometer (Monark 839E; Monark, Vansbro, Sweden). Participants were instructed to refrain from caffeine intake for at least 8 h and to avoid food consumption for at least 2 h prior to each test to standardize pre-exercise conditions. Respiratory gas exchange data were collected continuously using a breath-by-breath metabolic analyzer (MetaMax 3B; Cortex Biophysic, Leipzig, Germany). The system was calibrated before each test for ambient pressure, gas concentrations (O_2_ = 15.00%, CO_2_ = 5.00%), and volume (three L syringe), following manufacturer guidelines. Muscle oxygenation was monitored in real time using a near-infrared spectroscopy device (Moxy Monitor; Fortiori Design LLC, Hutchinson, MN, USA), with the sensor placed on the lateral vastus lateralis of the dominant leg (right leg in all participants). SmO_2_ was recorded throughout the trials. Capillary blood samples were collected immediately after the incremental test *via* fingertip puncture and analyzed for blood lactate concentration using a lactate analyzer (Biosen S-line Lab; EKF Diagnostics, Barleben, Germany).

### Incremental protocol

An incremental cycling test was conducted to determine each participant’s maximal oxygen uptake (VO_2_max) and power output at exhaustion. The protocol began with a 4-minute warm-up at 50 W, followed by a continuous increase in workload of 30 W per minute until volitional exhaustion. Participants maintained a self-selected cadence between 60 and 90 rpm, which was recorded and subsequently standardized across all future trials.

The test was terminated when the participant could no longer maintain the prescribed cadence for more than five consecutive seconds despite strong verbal encouragement. This termination criterion was consistently applied across all exhaustion-based protocols in the study. Exhaustion was confirmed when at least three of the following four criteria were met: (1) an increase in VO_2_ ≤ 150 mL/min despite increasing workload; (2) a post-exercise blood lactate concentration ≥ 8.0 mmol/L; (3) a respiratory exchange ratio (RER) ≥ 1.10; and (4) a peak heart rate ≥ 90% (208–0.7 × age).

The ventilatory thresholds VT_1_ and VT_2_—corresponding to the lactate threshold (LT) and the respiratory compensation point (RCP), respectively—were identified using ventilatory equivalents for oxygen and carbon dioxide (VE/VO_2_ and VE/VCO_2_), together with end-tidal gas analyses (PETO_2_ and PETCO_2_), following established methodological guidelines. Breath-by-breath VO_2_ data were processed using a 30-second moving average, and the highest averaged value was taken as VO_2_max (Keir et al., 2021).

### Submaximal constant-load testing protocol for AOD calculation

To determine AOD, each participant completed five submaximal constant-load cycling tests to establish the linear relationship between VO_2_ and power output. The target intensities ranged from 35% to 90% of each individual’s VO_2_max. Each test lasted 10 min, allowing sufficient time for VO_2_ to reach a steady state at each workload. Power levels were individually prescribed based on results from the incremental test and were administered in randomized order to minimize sequence effects.

Throughout each test, participants maintained a constant cadence (pre-selected and standardized between 60 and 90 rpm). VO_2_ was measured continuously using a breath-by-breath gas analyzer (MetaMax 3B; Cortex Biophysic, Leipzig, Germany). Steady-state VO_2_ was defined as the average value recorded during the final 2 min of each trial. A linear regression equation relating VO_2_ to power output was constructed using the five steady-state data points.

This regression equation was then used to estimate the theoretical oxygen demand during a subsequent supramaximal constant-load trial. AOD was calculated as the difference between the accumulated oxygen demand (predicted VO_2_ × duration) and the actual oxygen uptake measured during the supramaximal test, following the classical approach described by Noordhof, De Koning & Foster (2010).

### Priming exercise testing protocol

Participants completed three experimental sessions in a randomized crossover design, each involving a different priming strategy. Cadence was self-selected during the familiarization session, and each participant maintained the same individual cadence throughout all trials. Although the allowable range was 60–90 rpm, each athlete consistently used their pre-selected cadence, which was displayed on the ergometer in real time to ensure consistency. All other testing conditions—ergometer configuration, ambient temperature (18–21 °C), and time of day—were also held constant.

The three priming conditions were as follows (Goulding, Burnley & Wüst, 2023; Macdonald, Pedersen & Hughson, 1997): (1) LIPE, consisting of 6 min of cycling at 90% of VT_1_; (2) HIPE, performed at a workload equivalent to 0.4 × (Pmax − VT_1_) + VT_1_, corresponding to approximately 110% of critical power; and (3) LIPE-BFR, which replicated the LIPE workload and duration but incorporated external occlusion using an automated pneumatic cuff system (KAATSU Master; KAATSU Japan, Tokyo, Japan). Prior to each LIPE-BFR session, the participant’s individual arterial occlusion pressure (AOP) was assessed at rest using the device’s built-in detection algorithm. Pneumatic cuffs were placed around the proximal thighs and inflated to 70% of the measured AOP, beginning 30 s before cycling. The pressure was maintained throughout the 6-minute priming phase and released immediately upon completion. This individualized approach ensured consistent occlusion stimulus while minimizing the risk of over-restriction.

Each trial consisted of four consecutive phases: a 4-minute baseline phase at 20 W to stabilize resting VO_2_, a 6-minute priming phase following the condition-specific protocol, a 6-minute unloaded recovery phase at 20 W, and a 10-minute severe-intensity cycling bout to assess physiological responses. Trials were separated by at least 48 h to ensure full recovery.

Oxygen uptake was measured breath-by-breath using a metabolic analyzer (MetaMax 3B; Cortex Biophysic, Leipzig, Germany), interpolated to one Hz, and smoothed using a 5-second moving average. VO_2_ kinetics were modeled using nonlinear least-squares regression. During the priming phase, a mono-exponential model was applied:

\begin{eqnarray*}{\mathrm{VO}}_{2} \left( \mathrm{t} \right) ={\mathrm{VO}}_{2\mathrm{base}}+\mathrm{A}\times {e}^{ \left( - \left( t-\mathrm{TD} \right) /\tau \right) }, \end{eqnarray*}

where VO_2_ base is baseline VO_2_, A is the amplitude, TD is the time delay, and *τ* is the time constant.

For the 10-minute severe-intensity test, a bi-exponential model was used to capture both the primary and slow components:

\begin{eqnarray*}{\mathrm{VO}}_{2} \left( \mathrm{t} \right) ={\mathrm{VO}}_{2\mathrm{base}}+{\mathrm{A}}_{1}\times {e}^{ \left( - \left( t-{\mathrm{TD}}_{1} \right) /{\tau }_{1} \right) }+{\mathrm{A}}_{2}\times {e}^{ \left( - \left( t-{\mathrm{TD}}_{2} \right) /{\tau }_{2} \right) }. \end{eqnarray*}

Muscle oxygen saturation was continuously monitored *via* near-infrared spectroscopy (Moxy Monitor, Fortiori Design LLC, Hutchinson, MN, USA), with the sensor placed on the lateral vastus lateralis of the dominant leg (right leg in all participants). Six parameters were extracted to quantify tissue oxygenation: (1) SmO_2_ baseline, the mean SmO_2_ during the final 30 s of the baseline phase; (2) SmO_2_ min, the minimum SmO_2_ value recorded during the severe-intensity test; (3) SmO_2_ss, the mean SmO_2_ over the final 60 s of the test; (4) SmO_2_onset, the time to onset of deoxygenation, defined as the first sustained drop of ≥0.5% from baseline; (5) TimeSmO_2_min, the time from test onset to SmO_2_min; and (6) SmO_2_os, calculated as SmO_2_ss minusSmO_2_min, indicating the degree of reoxygenation. All SmO_2_ values were exported as device-derived % SmO_2_ estimates for subsequent analysis.

Capillary blood lactate concentration was measured at the following time points: before the priming exercise, 3 min and 5 min after the priming exercise, and 1 min, 4 min, and 7 min after the onset of the subsequent severe-intensity cycling bout. For analysis, Accumulated blood lactate was calculated as the maximum post-exercise BLa value across all sampling points minus the pre-exercise baseline value.

### Statistical analysis

All statistical analyses were conducted using SPSS version 27.0 (IBM Corp., Armonk, NY, USA). Data were first assessed for normality and screened for outliers. Mild outliers were adjusted using Windsorization, replacing extreme values with the nearest value within 1.5 times the interquartile range. Between-condition comparisons were analyzed using repeated-measures analysis of variance (RM-ANOVA), and results are reported using *P*-values and partial eta squared (*η*^2^) as the primary inferential and effect-size metrics. The sphericity assumption was assessed using Mauchly’s test; when violated, Greenhouse–Geisser–corrected results were reported. When significant main effects were observed, *post hoc* pairwise comparisons were performed with Bonferroni adjustment for multiple comparisons. Pearson’s product-moment correlation coefficients (r) were used to examine relationships between physiological variables. Correlation analyses were performed using difference scores (*e.g.*, HIPE − LIPE or HIPE − LIPE-BFR) to assess whether changes in one system (*e.g.*, VO_2_ kinetics) were associated with changes in another (*e.g.*, metabolic outcomes). A significance threshold of *P* < 0.05 was adopted for all tests.

## Results

### Acute physiological responses to different priming strategies

All outcome variables and statistical results are summarized in Table 2.

Compared with HIPE, both LIPE and LIPE-BFR induced significantly lower oxygen-uptake A and longer TD, whereas the Tau was significantly shorter in both low-intensity conditions (all *P* < 0.01). No significant differences were observed in VO_2_ baseline among the three conditions (*P* = 0.21).

Baseline SmO_2_ (SmO_2_ baseline) did not differ among conditions (*P* = 0.777). During exercise, HIPE produced significantly lower SmO_2_min than LIPE and LIPE-BFR, and LIPE-BFR showed lower SmO_2_min than LIPE (all *P* <  0.05), indicating greater muscle deoxygenation. SmO_2_ss showed the same pattern. SmO_2_on was longest in HIPE and significantly longer in LIPE-BFR than LIPE (*P* <  0.05). No significant differences were observed in Time SmO_2_ or SmO_2_os.

In terms of metabolic demand, AVO_2_, AOD, and ABLa were all significantly higher in HIPE than in LIPE and LIPE-BFR (all *P* < 0.01), confirming a substantially greater aerobic and anaerobic load in the high-intensity condition.

**Table 2:** Comparisons of oxygen uptake kinetics, tissue oxygenation, and metabolic responses under different priming exercise conditions.

	HIPE	LIPE	LIPE-BFR	Sig.	Partial *η*^2^
A	25.10 ± 2.76	12.91 ± 3.59^*^	13.73 ± 3.42^*^	<0.001	0.758
TD	4.01 ± 3.65	9.80 ± 4.34^*^	8.98 ± 4.43^*^	0.001	0.292
Tau	68.59 ± 11.08	45.68 ± 12.22^*^	51.43 ± 13.53^*^	<0.001	0.405
VO_2_ baseline	12.67 ± 1.07	11.94 ± 1.53	11.93 ± 1.30	0.21	0.069
SmO_2_ baseline	64.45 ± 2.12	64.59 ± 2.32	64.02 ± 2.53	0.788	0.011
SmO_2_min	48.69 ± 5.34	56.26 ± 3.93^*^	52.52 ± 5.65^*^^#^	0.001	0.298
SmO_2_ss	49.99 ± 4.89	58.56 ± 3.27^*^	54.89 ± 4.21^*^^#^	<0.001	0.440
TimeSmO_2_	280.47 ± 98.10	220.81 ± 130.27	190.50 ± 137.84	0.145	0.088
SmO_2_on	15.83 ± 3.72	8.21 ± 3.56^*^	11.33 ± 4.30^*^^#^	<0.001	0.420
SmO_2_os	−1.46 ± 1.47	−2.30 ± 2.08	−2.15 ± 1.72	0.389	0.044
AVO_2_	12,264.97 ± 1,856.90	8,603.55 ± 1,730.32^*^	8,706.00 ± 1,781.68^*^	<0.001	0.491
AOD	1,988.54 ± 678.70	1,080.60 ± 540.39^*^	957.56 ± 568.11^*^	<0.001	0.386
ABLa	5.08 ± 2.21	3.13 ± 1.40^*^	2.69 ± 2.00^*^	0.002	0.241

**Notes.**

Values are presented as mean ± SD.

A amplitude TD time delay Tau time constant VO_2_ baseline baseline oxygen uptake SmO_2_ muscle oxygen saturation AVO_2_ accumulated oxygen uptake AOD accumulated oxygen deficit ABLa Accumulated blood lactate concentration

* *P* < 0.05 *vs.* HIPE.

# *P* < 0.05 *vs.* LIPE.

### Correlations between oxygen uptake kinetics, muscle oxygen saturation, and metabolic load

**Figure 1:**
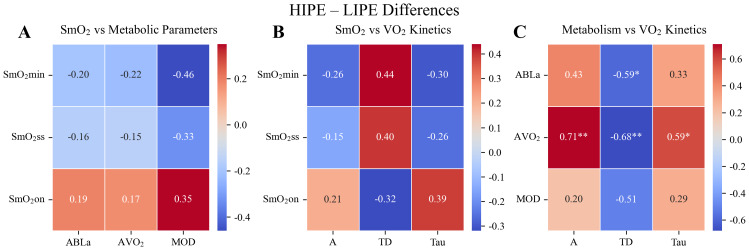
Pearson correlation heatmaps of variable differences between HIPE and LIPE. (A) Correlations between muscle oxygen saturation (SmO_2_min, SmO_2_ss, SmO_2_on) and metabolic parameters, including Accumulated blood lactate concentration(ABLa). accumulated oxygen uptake (AVO_2_), and accumulated oxygen deficit (AOD). (B) Correlations between tissue oxygenation indices and oxygen uptake (VO_2_) kinetics parameters, including amplitude (A), time delay (TD), and time constant (*τ*). (C) Correlations between metabolic parameters and VO_2_ kinetics parameters. Color gradients represent the strength and direction of correlations (red = positive, blue = negative). Asterisks indicate statistical significance (^∗^*P* < 0.05, ^∗∗^*P* < 0.01).

**Figure 2:**
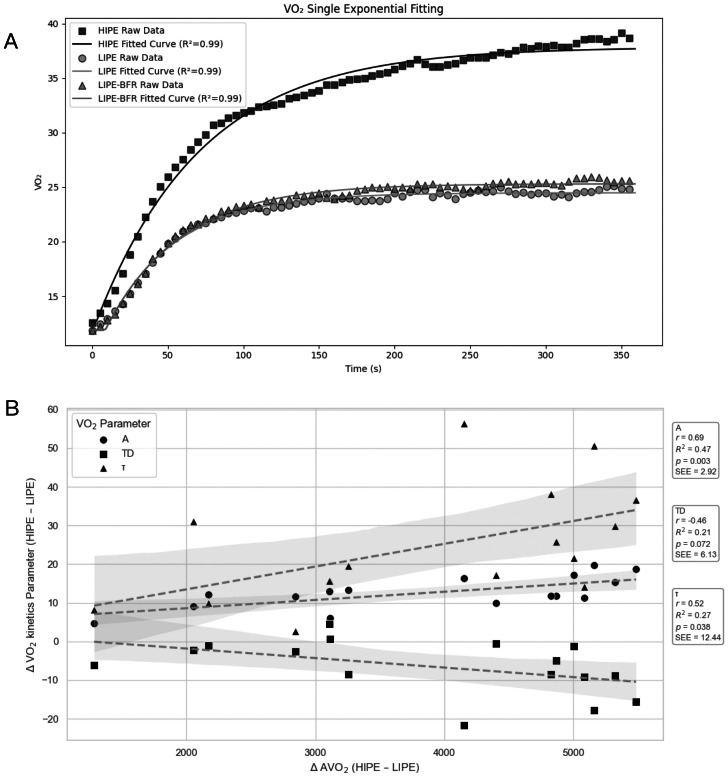
VO_2_ kinetics responses and their associations with VO_2_ accumulation under different priming exercise conditions. (A) Pulmonary oxygen uptake (VO_2_) time courses for high-intensity priming exercise, low-intensity priming exercise, and low-intensity priming exercise with blood flow restriction. Raw VO_2_ data are shown together with single-exponential model fits, illustrating distinct VO_2_ kinetic profiles across conditions. (B) Relationships between between-condition differences in accumulated VO_2_ (Δ AVO_2_; high-intensity priming exercise minus low-intensity priming exercise) and corresponding changes in VO_2_ kinetic parameters, including amplitude (ΔA), time delay (ΔTD), and time constant (Δ*τ*). Each data point represents an individual participant. Linear regression lines with 95% confidence intervals are shown to illustrate the direction and strength of associations.

Significant associations were identified between metabolic outcomes and oxygen uptake kinetics in the comparison of HIPE and LIPE (Figs. 1A–1C). AVO_2_ demonstrated a strong positive correlation with the amplitude of the oxygen uptake response (*r* = 0.71, *P* < 0.01), and significant negative correlations with both the time delay (r = −0.66, *P* < 0.01) and the time constant (Tau) (r = −0.59, *P* < 0.05). These findings suggest that individuals exhibiting a more rapid and pronounced VO_2_ kinetics response also experienced greater overall metabolic demand. These statistical results were corroborated by the time-course profiles of VO_2,_ which showed a steeper and faster rise in the HIPE group compared to more gradual increases observed in LIPE and LIPE-BFR (Figs. 2A and 2B). No other relationships reached statistical significance (Figs. 1A–1C).

**Figure 3:**
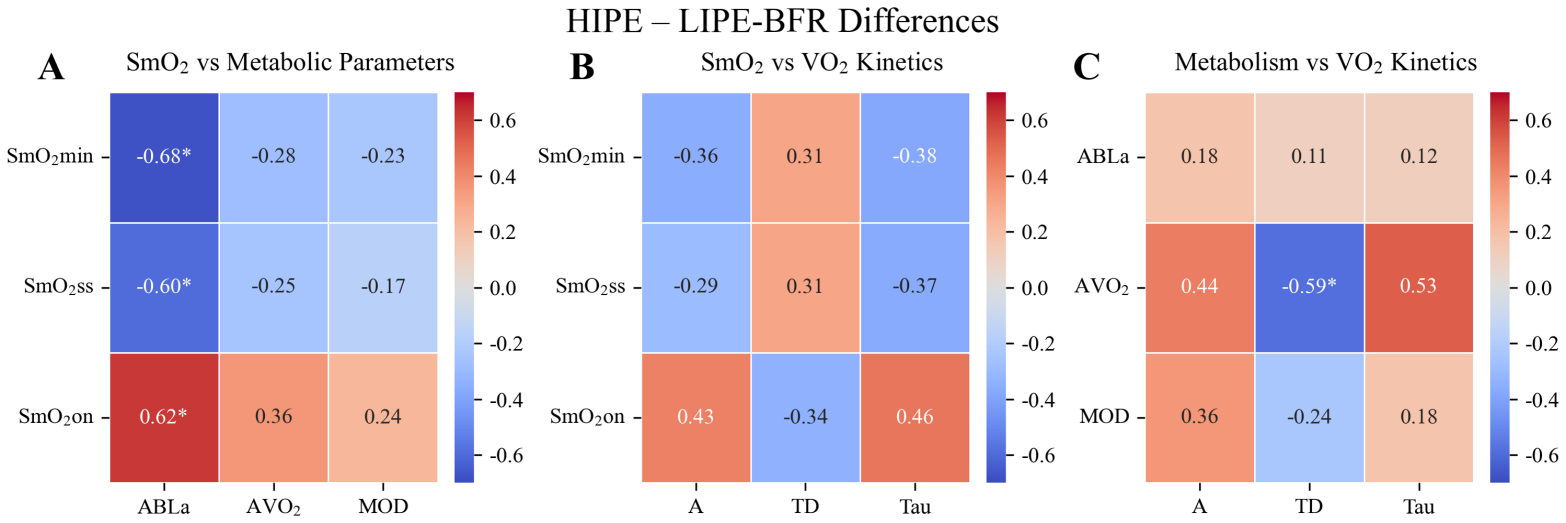
Pearson correlation heatmaps of variable differences between HIPE and LIPE-BFR. (A) Correlations between muscle oxygen saturation (SmO_2_min, SmO_2_ss, SmO_2_on) and metabolic parameters (ABLa, AVO_2_, AOD). (B) Correlations between tissue oxygenation indices and pulmonary VO_2_ kinetics parameters (A, TD, *τ*). (C) Correlations between metabolic parameters and pulmonary VO_2_ kinetics parameters. Color gradients represent the direction and magnitude of the correlations (red = positive, blue = negative). Asterisks indicate statistically significant correlations (^∗^*P* < 0.05; ^∗∗^*P* < 0.01).

**Figure 4:**
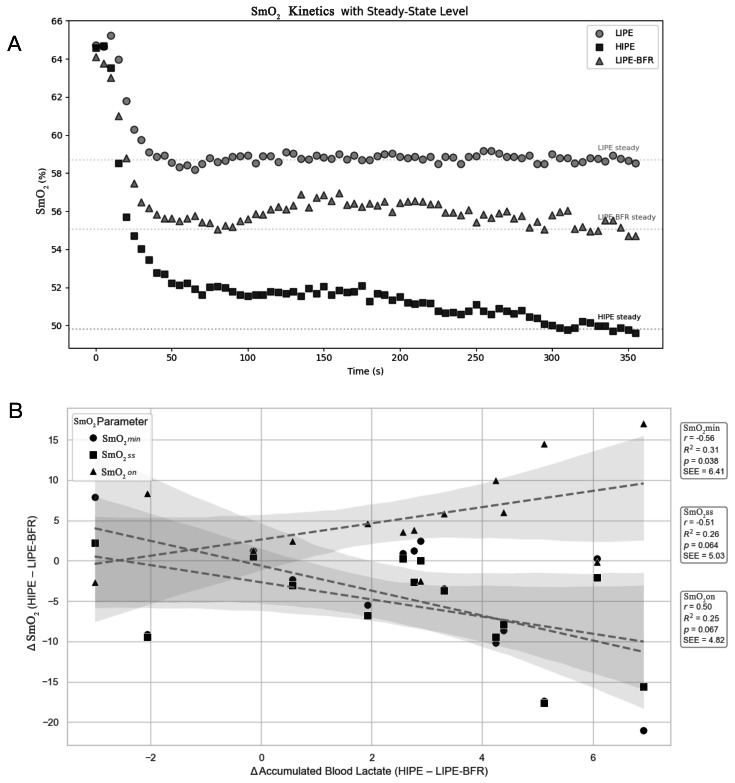
Muscle oxygen saturation (SmO_2_) responses and their associations with blood lactate differences between HIPE and LIPE-BFR. (A) Time course of the muscle oxygen saturation (SmO_2_, %) during the priming bout across the three conditions (LIPE, HIPE, and LIPE-BFR). Symbols denote condition-specific SmO_2_ values over time (s), and horizontal dotted lines indicate the corresponding steady-state SmO_2_ level for each condition. (B) Relationships between between-condition differences in tissue oxygenation and lactate accumulation. The *y*-axis shows Δ SmO_2_ (HIPE - LIPE-BFR) for three SmO_2_-derived indices (SmO_2_min, SmO_2_ss, SmO_2_on), and the *x*-axis shows the difference in accumulated blood lactate (HIPE - LIPE-BFR). Each point represents an individual participant. Dashed lines indicate the fitted linear regression for each SmO_2_ index, with shaded 95% confidence bands. Pearson’s r, R^2^, *p* value, and the standard error of estimate (SEE) are reported in the inset boxes.

In the comparison of HIPE and LIPE-BFR, the strongest correlations emerged between tissue oxygenation indices and accumulated blood lactate (Figs. 3A–3C). SmO_2_min (r = −0.66, *P* < 0.05) and SmO_2_ss (r = −0.60, *P* < 0.05) were both negatively correlated with ABLa, indicating that greater muscle deoxygenation was associated with higher lactate accumulation. In contrast, SmO_2_on was positively correlated with ABLa (*r* = 0.62, *P* < 0.05), suggesting that delayed onset of deoxygenation was also linked to increased metabolic stress. These patterns were consistent with the SmO_2_ dynamic trajectories, where HIPE induced an earlier and deeper decline in oxygenation, while the LIPE-BFR group exhibited an intermediate response (Figs. 4A and 4B). Additionally, AVO_2_ was negatively correlated with TD (r = −0.59, *P* < 0.05). Other correlations were not statistically significant (Figs. 3A–3C).

### Physiological responses during subsequent severe-intensity exercise

All outcome variables and statistical results are summarized in Table 3. No statistically significant differences were observed among the three groups (HIPE-HIE, LIPE-HIE, and BFR-HIE) in oxygen uptake kinetics parameters, including the primary (A_1_) and slow component (A_2_) amplitudes, time delays (TD_1_, TD_2_), and time constants (Tau_1_, Tau_2_) (all *P* >  0.2). Tissue oxygenation indices—SmO_2_ baseline, SmO_2_ min, SmO_2_ss, TimeSmO_2_, SmO_2_ on, and SmO_2_ os—also showed no significant differences across conditions (all *P* > 0.1).

**Table 3:** Physiological responses during severe-intensity exercise following different priming exercise conditions.

	HIPE-HIE	LIPE-HIE	BFR-HIE	Sig.	Partial *η*^2^
A_1_	19.03 ± 3.16	18.66 ± 3.20	18.94 ± 2.91	0.940	0.003
TD_1_	9.49 ± 3.01	11.58 ± 3.66	10.94 ± 4.92	0.312	0.052
Tau_1_	31.99 ± 8.01	34.54 ± 6.84	31.47 ± 9.32	0.526	0.029
A_2_	7.60 ± 3.17	9.44 ± 2.75	9.10 ± 3.21	0.207	0.069
TD_2_	42.91 ± 19.33	48.39 ± 31.04	54.44 ± 37.95	0.573	0.025
Tau_2_	263.11 ± 62.27	263.94 ± 51.60	263.24 ± 47.87	0.999	<0.001
VO_2_ Baseline	15.65 ± 1.82	13.48 ± 1.45	13.58 ± 1.51	<0.001	0.296
A_1_/(A_1_+A_2_)	0.28 ± 0.10	0.33 ± 0.08	0.32 ± 0.09	0.275	0.057
SmO_2_ baseline	68.39 ± 3.77	66.53 ± 3.02	65.99 ± 3.98	0.177	0.079
SmO_2_min	47.58 ± 6.70	49.23 ± 5.91	46.03 ± 7.88	0.447	0.038
SmO_2_ss	48.81 ± 6.56	50.40 ± 6.00	47.61 ± 7.18	0.512	0.031
TimeSmO_2_	420.81 ± 202.89	429.31 ± 207.08	397.94 ± 204.33	0.905	0.004
SmO_2_on	19.51 ± 9.54	17.30 ± 6.17	17.46 ± 9.25	0.714	0.047
SmO_2_os	−1.16 ± 1.00	−1.17 ± 0.84	−1.38 ± 1.21	0.781	0.033
AVO_2_	23,855.41 ± 4,304.42	22,746.32 ± 4,062.43	23,073.83 ± 4,080.28	0.742	0.013
AOD	1,305.77 ± 636.13	1,925.42 ± 818.81	1,867.97 ± 869.20	0.057	0.122
ABLa	7.41 ± 3.18	7.69 ± 1.98	7.09 ± 2.60	0.81	0.009

**Notes.**

Values are expressed as mean ± SD.

A1, A2 primary and slow component amplitudes TD1, TD2 time delays Tau1, Tau2 time constants VO_2_ Baseline resting oxygen uptake SmO_2_ muscle oxygen saturation AVO_2_ accumulated oxygen uptake AOD accumulated oxygendeficit ABLa Accumulated blood lactate concentration

Similarly, no group differences were found in AVO_2_, AOD, or BLa, with *P* values of 0.742, 0.057, and 0.81, respectively. The only variable showing a statistically significant difference was baseline oxygen uptake (VO_2_ Baseline), which was higher in the HIPE-HIE group compared to both LIPE-HIE and BFR-HIE (*P* < 0.001).

## Discussion

This randomized crossover study systematically compared the effects of three priming exercise strategies (HIPE, LIPE, and LIPE-BFR) on VO_2_ kinetics, SmO_2_, and metabolic responses during severe-intensity cycling in moderately trained anaerobic athletes. A key finding was that, although the three conditions elicited distinct acute physiological responses during the priming phase, including changes in oxygen uptake parameters, muscle oxygenation indices (SmO_2_min and SmO_2_ss), and metabolic indicators such as AVO_2_, AOD, and ABLa, their residual effects on VO_2_ kinetics and metabolic load during the subsequent exercise bout were largely comparable. These findings suggest that, under the present protocol configuration, the acute physiological activation induced by priming was not associated with differentiated residual responses in the subsequent bout. In particular, the 6-min recovery interval and the relatively low-intensity BFR stimulus may warrant further investigation when designing priming strategies for anaerobic athletes.

The three priming strategies in this study exhibited distinct physiological characteristics during the intervention phase. Compared to HIPE, both LIPE and LIPE-BFR resulted in lower A and longer TD, indicating reduced overall metabolic load and oxygen mobilization. Meanwhile, HIPE induced the greatest muscle deoxygenation, as evidenced by the lowest SmO_2_min and SmO_2_ss values. LIPE-BFR showed intermediate deoxygenation levels between HIPE and LIPE. This pattern suggests that LIPE-BFR may increase local metabolic stress under low mechanical load, the extent of deoxygenation is still less than that induced by HIPE. These findings confirm the efficacy of BFR in eliciting local metabolic activation, consistent with results reported by Lixandrao et al. (2018). The lower SmO_2_min and SmO_2_ss observed in LIPE-BFR compared to LIPE further support the role of BFR in simulating high metabolic load under low-intensity conditions (Patterson et al., 2019). SmO_2_min and SmO_2_ss reflect primarily end-point characteristics of muscle oxygenation. From a kinetics perspective, parameters such as the rate and timing of deoxygenation and reoxygenation may provide additional insight into the dynamic matching between local O_2_ delivery and O_2_ utilization during priming transitions (Dunst et al., 2023; Ferrari, Muthalib & Quaresima, 2011). However, this enhancement in local deoxygenation did not translate into a clear improvement in systemic metabolic outcomes during the subsequent high-intensity exercise. In addition, the correlation analyses conducted during the priming phase further clarified these acute physiological patterns: deeper muscle deoxygenation (lower SmO_2_min and SmO_2_ss) was linked to higher lactate accumulation, and faster VO_2_ kinetics profiles (larger A and shorter TD/Tau) were associated with greater metabolic demand. These relationships support an association among oxygen uptake kinetics, SmO_2_-derived indices, and metabolic-load markers during the priming exercise.

Contrary to previous findings suggesting that priming exercise may enhance subsequent VO_2_ kinetics during high-intensity exercise (Goulding, Burnley & Wüst, 2023; Bailey et al., 2009), this study did not observe such a carry-over effect. Gerbino, Ward & Whipp (1996) emphasized that improvements in VO_2_ kinetics following priming are highly dependent on the degree of cardiovascular activation and the duration of the recovery period. The 6-minute recovery employed in this study may have been insufficient to allow VO_2_ and metabolic byproducts to return to baseline, thereby masking any potential priming effect. In fact, a significantly elevated baseline VO_2_ was observed in the HIPE condition at the onset of the subsequent severe-intensity exercise, supporting the concept of a “residual effect” proposed by Burnley et al. Jones & Poole (2005), in which incomplete recovery may attenuate potential priming benefits. Rossiter et al. (2002) further noted that a brief recovery period may not sufficiently reduce lactate accumulation and hydrogen ion concentration induced by prior exercise, which could impair muscle contractile efficiency and metabolic responses during the subsequent bout. In line with these observations, the lack of significant reductions in blood lactate and AOD during the follow-up HIIT session in this study suggests that the residual accumulation of metabolic byproducts may have contributed to the absence of a detectable priming benefit.

Beyond the issue of insufficient recovery duration, fatigue induced by the priming exercise itself, whether local or systemic, may also attenuate subsequent performance improvements (Burnley & Jones, 2007). For example, Burnley, Doust & Jones (2005) reported that under short recovery conditions, the metabolic burden caused by high-intensity priming may exceed its intended metabolic activation effects, thereby impairing muscle contractile efficiency during the following exercise. Ferguson et al. (2007) further suggested that if priming leads to excessive metabolic stress, such as elevated concentrations of lactate, hydrogen ions, and inorganic phosphate, these metabolites may persist during the brief recovery period and hinder effective muscle fiber recruitment and metabolic transitions during subsequent efforts. This mechanism may also explain why VO_2_ kinetics parameters showed neither improvement nor deterioration following the priming interventions.

From the perspective of oxygen delivery and utilization matching, Mortensen et al. (2008) indicated that acute blood flow redistribution and vasodilation during priming may temporarily enhance local oxygen supply. However, these changes may not be sustained after a short recovery period, thereby limiting the functional transfer of priming effects. In addition, competition for systemic oxygen delivery resulting from regional redistribution may further reduce metabolic efficiency during the subsequent exercise, potentially accounting for the absence of kinetics improvements (Koga et al., 2014). Therefore, the combined impact of priming-induced fatigue and residual metabolic stress may have offset any performance-enhancing effects under the current recovery conditions, which could be one of the main reasons why this study did not observe sustained priming effects.

Although no significant changes were observed in VO_2_ kinetics parameters such as A_1_, Tau_1_, and TD_1_ in this study, the elevated VO_2_ baseline following priming is more likely reflective of incomplete recovery rather than enhanced oxygen delivery mechanisms (Burnley, Doust & Jones, 2005). Jones, Koppo & Burnley (2003) proposed that priming may optimize metabolic responses primarily by increasing the primary amplitude rather than altering Tau or TD. In this study, a higher VO_2_ baseline was similarly observed in the HIPE condition, although it did not translate into improvements in kinetics parameters. This may highlight the importance of recovery strategy, as the priming effect may only emerge when sufficient recovery time is provided to allow physiological indices to return to baseline levels. Furthermore, attenuation of the VO_2_ slow component is typically associated with improved motor unit recruitment patterns and enhanced oxygen delivery efficiency (Xu & Rhodes, 1999). The absence of significant changes in the slow component in this study may indicate that such microcirculatory or neuromuscular adaptations were not effectively elicited by the priming interventions. Previous studies have suggested that the VO_2_ slow component reflects a progressive increase in oxygen demand during sustained high-intensity exercise and is closely related to the gradual recruitment of type II muscle fibers and the decline in metabolic efficiency due to fatigue (Jones et al., 2011). Theoretically, appropriate priming could facilitate better matching between oxygen delivery and utilization, reducing excessive type II fiber recruitment during the subsequent task and thereby decreasing the magnitude of the slow component (Burnley & Jones, 2007; Ferguson et al., 2007).

This outcome may be related to the relatively low absolute intensity and moderate occlusion pressure used (90% VT_1_, 70% AOP), which may not have reached the threshold necessary to induce a substantial systemic metabolic load. These findings support the view of Patterson et al. (2019), who emphasized that the effectiveness of BFR interventions is highly dependent on individualized optimization, particularly in populations with high levels of training adaptation where a greater metabolic challenge may be required. Furthermore, Manini & Clark (2009) proposed that individual variability in vascular responsiveness during BFR may significantly influence systemic metabolic outcomes. This mechanistic variability may explain the substantial inter-individual differences in response to BFR observed in this study. The impact of individual differences on BFR effectiveness should not be overlooked. Cook et al. (2010) reported considerable variability in vascular compliance and sensitivity during blood flow restriction training, which can directly affect the extent of muscle hypoxia and the subsequent metabolic responses. Such variability may be particularly relevant in trained athletes with relatively efficient vascular regulation, potentially limiting the systemic impact of a mild BFR priming stimulus.

This study has several limitations. First, although distinct local metabolic responses were observed during the priming phase, these responses did not appear to produce differentiated residual effects on oxygen uptake kinetics after the short recovery period used in this study. In addition, because a passive control condition (no priming) was not included, the present findings should be interpreted primarily as comparisons among priming protocols rather than as evidence for the overall effectiveness of priming. Second, direct assessments of neuromuscular recruitment patterns and microvascular kinetics were not included, although both may contribute to the expression of priming effects. Future studies may benefit from integrating near-infrared spectroscopy with neuromuscular electrophysiological measurements to better characterize the underlying mechanisms. Third, although both male and female athletes were included, the sample size was insufficient to support sex-based subgroup analyses and may have limited sensitivity to detect small-to-moderate between-condition effects, warranting cautious interpretation of statistically nonsignificant findings. In addition, adipose tissue thickness at the NIRS site was not measured, which may have contributed to inter-individual variability in SmO_2_ estimates. Accordingly, relative time-course features of SmO_2_ responses may provide a more robust basis for interpretation than absolute values alone. Future research should further examine alternative priming configurations, including recovery duration, occlusion pressure, and progressive priming, and extend comparisons across different exercise intensities, athlete populations, and larger samples.

## Conclusions

In summary, although the three priming strategies elicited distinct local physiological responses during the priming phase, their residual effects on oxygen uptake kinetics and metabolic load during subsequent severe-intensity exercise were largely comparable. Future research should further examine alternative priming configurations, particularly with respect to recovery duration, occlusion pressure, and priming intensity.

##  Supplemental Information

10.7717/peerj.21221/supp-1 Supplemental Information 1 Dataset of oxygen uptake kinetics, muscle oxygenation, and metabolic load under different priming exercise intensities Table 1: The baseline characteristics of all participants; Table 2: Group comparisons of oxygen uptake kinetics, tissue oxygenation, and metabolic responses under different exercise conditions; Table 3: Summary of the physiological responses during high-intensity exercise following different priming exercise conditions (HIPE, LIPE, and LIPE-BFR). The variables include pulmonary oxygen uptake kinetics (A, TD, *τ*, VO_2_ baseline), muscle oxygen saturation indices (*e.g.*, SmO_2_min, SmO_2_ss), blood lactate concentrations, accumulated oxygen difference (AVO_2_), and accumulated oxygen deficit (AOD), with each data point reflecting an individual measurement under a specific condition.

## Additional Information and Declarations

### Data Availability

The following information was supplied regarding data availability: The raw data is available in the Supplementary File.
